# Health Tract, No. 41

**Published:** 1865

**Authors:** 


### HEALTH TRACT, No. 41.

---

### SICK HEAD-ACHE

Is sickness at stomach, a tendency to vomit, combined with pain in some part of the head, generally the left side. It is caused by there being too much bile in the system, from the fact that this bile is manufactured too rapidly, or is not worked out of the system fast enough by steady, active exercise. Hence sedentary persons, those who do not walk about a great deal, but are seated in the house nearly all the time, are almost exclusively the victims of this distressing malady. It usually begins soon after waking up in the morning, and lasts a day or two or more. There are many causes; the most frequent is, derangement of the stomach by late and hearty suppers; by eating too soon after a regular meal, (five hours should, at least, intervene;) eating without an appetite; forcing food; eating after one is conscious of having had enough; eating too much of any favorite dish; eating something which the stomach can not digest, or sour stomach. Any of these things may induce sick head-ache; all of them can be avoided. Over-fatigue or great mental emotion of any kind, or severe mental application, have brought on sick head-ache, of the most distressing character, in an hour; it is caused by indulgence in spirituous liquors. When a person has sick head-ache, there is no appetite; the very sight of food is hateful; the tongue is furred; the feet and hands are cold, and there is a feeling of universal discomfort, with an utter indisposition to do any thing whatever. A glass of warm water, into which has been rapidly stirred a heaping tea-spoon each of salt and kitchen mustard, by causing instantaneous vomiting, empties the stomach of the bile or undigested sour food, and a grateful relief is often experienced on the spot; and rest, with a few hours of sound, refreshing sleep, completes the cure, especially if the principal part of the next day or two is spent in mental diversion and out-door activities, not eating an atom of food (but drinking freely of cold water or hot teas) until you feel as if a piece of plain, cold bread and butter would "taste really good." Nine times in ten the cause of sick head-ache is in the fact, that the stomach was not able to digest the food last introduced into it, either from its having been unsuitable, or excessive in quantity. When the stomach is weak, a spoonful of the mildest, blandest food would cause an attack of sick head-ache, when ten times the amount might have been taken in health, not only with impunity but with positive advantage.

Those who are "subject to sick head-ache" eat too much and exercise too little, and have cold feet and constipation. (See Health Tracts Nos. 21 and 22.) A diet of cold bread and butter, and ripe fruits or berries, with moderate continuous exercise in the open air, sufficient to keep up a very gentle perspiration, would, of themselves, cure almost every case within thirty-six hours. Two teaspoonfuls of pulverized charcoal, stirred in half a glass of water, and drank, generally gives instant relief.

